# A Meta-Analysis and Indirect Comparison of Endothelin A Receptor Antagonist for Castration-Resistant Prostate Cancer

**DOI:** 10.1371/journal.pone.0133803

**Published:** 2015-07-20

**Authors:** Ping Qi, Ming Chen, Li-xiu Zhang, Rui-xia Song, Zhen-hua He, Zhi-ping Wang

**Affiliations:** 1 Institute of Urology, The Second Hospital of Lanzhou University, Lanzhou 730030, China; 2 Key Laboratory of Urological Diseases in Gansu Province, Lanzhou 730030, China; 3 Department of Clinical Laboratory, The Second Hospital of Lanzhou University, Lanzhou 730030, China; 4 Department of Urology, GanSu Provincial Hospital of Traditional Chinese Medicine, Lanzhou 730050, China; 5 Department of Neurosurgery, The Second Hospital of Lanzhou University, Lanzhou 730030, China; UC Davis Comprehensive Cancer Center, UNITED STATES

## Abstract

**Background:**

Endothelin A (ET-A) receptor antagonists including zibotentan and atrasentan, have been suggested as a treatment for castration-resistant prostate cancer (CRPC). Our aim was to conduct a meta-analysis and indirect comparison to assess the efficacy and safety of ET-A receptor antagonists for treatment of CRPC.

**Methods:**

We systematically searched PubMed, EMBASE, the Cochrane Library, and Web of Science from inception to November 2014 to identify randomized controlled trials (RCTs) which assessed ET-A receptor antagonists for treatment of CRPC. Meta-analysis was conducted by STATA version 12.0 software.

**Results:**

Eight RCTs were identified, involving 6,065 patients. The results of direct comparison showed that compared with placebo, there was no statistically significant difference in the improvement of progression-free survival (PFS), overall survival (OS), time to disease progression (TTP), and total adverse events (AEs) with ET-A receptor antagonist treatment for CRPC. The results of ET-A receptor antagonists plus docetaxel versus docetaxel alone were similar. The indirect comparisons showed that there were no significant differences between zibotentan plus docetaxel versus atrasentan plus docetaxel when compared with docetaxel alone or zibotentan versus atrasenta compared with placebo in the improvement of PFS, OS, TTP, and total adverse events.

**Conclusions:**

There were no significant benefits for ET-A receptor antagonists with or without docetaxel in the improvement of PFS, OS, TTP, and overall AEs. And there were no significant differences between zibotentan and atrasentan. Single-agent docetaxel should remain as one of the standard treatments.

## Introduction

Prostate cancer (PCa) is the most common type of malignant neoplasm in men in the western world. The American Cancer Society (ACS) estimates that there will be 220,800 new cases of PCa and an estimated 27,540 people will die of this disease in the United States in 2015. Associated mortality rates in many developed countries have been reduced due to improvements in treatment[1]. By contrast, the incidence of PCa and its related mortality rates are increasing in Asian as well as Central and East European countries [1,2,3].

PCa is a hormonally-sensitive disease [4] and androgen deprivation therapy (ADT) is the most common treatment regime[5]. Unfortunately, the majority of prostate cancers become hormone insensitive and eventually develop metastases [6]. Castration-resistant prostate cancer (CRPC) has a poor prognosis with limited therapeutic options[6]. Previous meta-analysis showed that docetaxel-based combination chemotherapy for patients with CRPC had good results[7], however, as a chemotherapy regimen it has an associated toxicity [8]. New treatment options for patients with CRPC are needed to improve survival while avoiding the toxicity associated with chemotherapy[8].

In recent years, endothelin A (ET-A) receptor antagonists have been suggested as a treatment for CRPC. Zibotentan (ZD4054) is an oral and selective ET-A receptor antagonist in development. Atrasentan (ABT-627) is another orally bioavailable selective ET-A receptor antagonist that inhibits ET-1 activity [9]. Some randomized controlled trials (RCTs) of ET-A receptor antagonists for the treatment of CRPC are currently available, however the results of those studies have been inconsistent. Also the efficacy differences between zibotentan and atrasentan have not been compared. A systematic review by Shao N et al.[9] comparing ET-A receptor antagonists to placebo for CRPC suggested ET-A receptor antagonists are an attractive option for such patients. However, only four studies were included in the analysis, and the comparison of ET-A receptor antagonists and docetaxel was not performed. In addition, the efficacy differences between zibotentan and atrasentan were not investigated. Our study aims to systematically compare the efficacy and safety of ET-A receptor antagonists and placebo or docetaxel. Secondly, an indirect comparison will be conducted to assess the efficacy and safety of different ET-A receptor antagonist-based regimens. To our knowledge, there has been no prior meta-analysis comparing these two drugs.

## Materials and Methods

### Inclusion criteria

RCTs that compared ET-A receptor antagonists to other agents for CRPC were considered eligible. The selected RCTs met the following inclusion criteria: (1) Participants ≥ 18 years old, histologically or cytologically confirmed CRPC (including metastatic and non-metastatic forms). (2) RCT or “random” was mentioned in groups. (3) Outcomes: the primary endpoints were progression-free survival (PFS) and overall survival (OS). Secondary endpoints were time to disease progression (TTP) and adverse events (AEs).

Exclusion criteria were: (1) CRPC patients with brain metastases, active infection, and clinically significant ascites or pleural effusion (2) the intervention was not ET-A receptor antagonists. (3) animal studies, case-reports, reviews or meta-analyses/ systematic reviews and (4) abstracts or letters to the journal editors.

Two reviewers independently screened studies according to pre-specified inclusion and exclusion criteria. Disagreements were resolved in consultation with a third reviewer.

### Search strategy

All relevant RCTs were identified by searching PubMed (1966–2014.11), EMBASE.com (1974–2014.11), Cochrane Library (CENTRAL, Issue 11 of 12, November 2014), and Web of Science (2000–2014.11). We combined MeSH terms and free terms in all the search strategies and adjusted accordingly for the different databases, using the following terms: prostatic cancer, prostatic tumor, prostatic carcinoma, prostatic neoplasm, prostate cancer, prostate tumor, prostate carcinoma, prostate neoplasm, castration resistant, hormone refractory, androgen independent, androgen insensitive, androgen resistant, endothelin A receptor antagonist, zibotentan, ZD4054, atrasentan, ABT-627, randomized controlled trials, random*. In addition to electronic search for relevant studies, we also screened the references of included studies and reviews to look for potentially eligible studies. The search strategy was independently conducted by two reviewers. And the search strategy of PubMed was as follows:

#1 "Castration Resistant" OR "Hormone Refractory" OR "Androgen Independent" OR "Androgen Insensitive" OR "Androgen Resistant"[Title/Abstract]
#2 prostatic cancer* OR prostatic tumor* OR prostatic carcinoma* OR prostatic neoplasm* OR prostate cancer* OR prostate tumor* OR prostate carcinoma* OR prostate neoplasm*[Title/Abstract] OR "Prostatic Neoplasms"[Mesh]
#3 #1 AND #2
#4 "endothelin A receptor antagonist"[Title/Abstract] OR zibotentan[Title/Abstract] OR ZD4054[Title/Abstract] OR atrasentan[Title/Abstract] OR ABT-627[Title/Abstract]
#5 "atrasentan" [Supplementary Concept]) OR "ZD4054" [Supplementary Concept]
#6 #4 OR #5
#7 random* OR randomized controlled trial* OR randomized trial* OR Randomized Controlled Trial[ptyp] OR "Randomized Controlled Trials as Topic"
#8 #3 AND #6 AND #7

### Data extraction and quality assessment

A standard data extraction form was designed to include authors, publication year, intervention, number in the sample, multicenter, journal, median age, median PSA, median PFS, median OS, and outcome, etc. The methodological quality was assessed according to the Cochrane Handbook version 5.1.0[10] including adequate sequence generation, adequate allocation concealment, blinding, incomplete outcome data addressed, and freedom from selective reporting. The judgments for each entry involve assessing the risk of bias as ‘low risk’, as ‘high risk’, or as ‘unclear risk’. Data extraction and quality assessment was performed by two independent reviewers, and disagreements were resolved by consensus.

### Statistical analysis

Calculation was done of the overall hazard ratio (HR) for PFS, OS, and TTP. The odds ratio (OR) for grades 3 or 4 AEs were calculated. The Chi-square statistic was used to assess the heterogeneity between trials with *I* ^*2*^ less than 50% and *P*-value greater than 0.10 suggesting that there was no statistical heterogeneity. A fixed effects model was used for meta-analysis. Publication bias was examined using Begg’s funnel plot. Sensitivity analysis was performed to identify influence of the study regarding overall effective size. *P*-value less than 0.05 was considered significant. Direct comparisons were calculated using Stata version 10.0 software (Stata Corporation, College Station, Texas, USA). Indirect comparisons were calculated using Indirect Treatment Comparison (ITC) software (Canadian Agency for Drugs and Technologies in Health, Canada). The reporting of this meta-analysis adhered to the Preferred Reporting Items for Systematic Review and Meta-Analysis (PRISMA) statement (S1 PRISMA Checklist)[11].

## Results

### Search results

**Fig 1:**
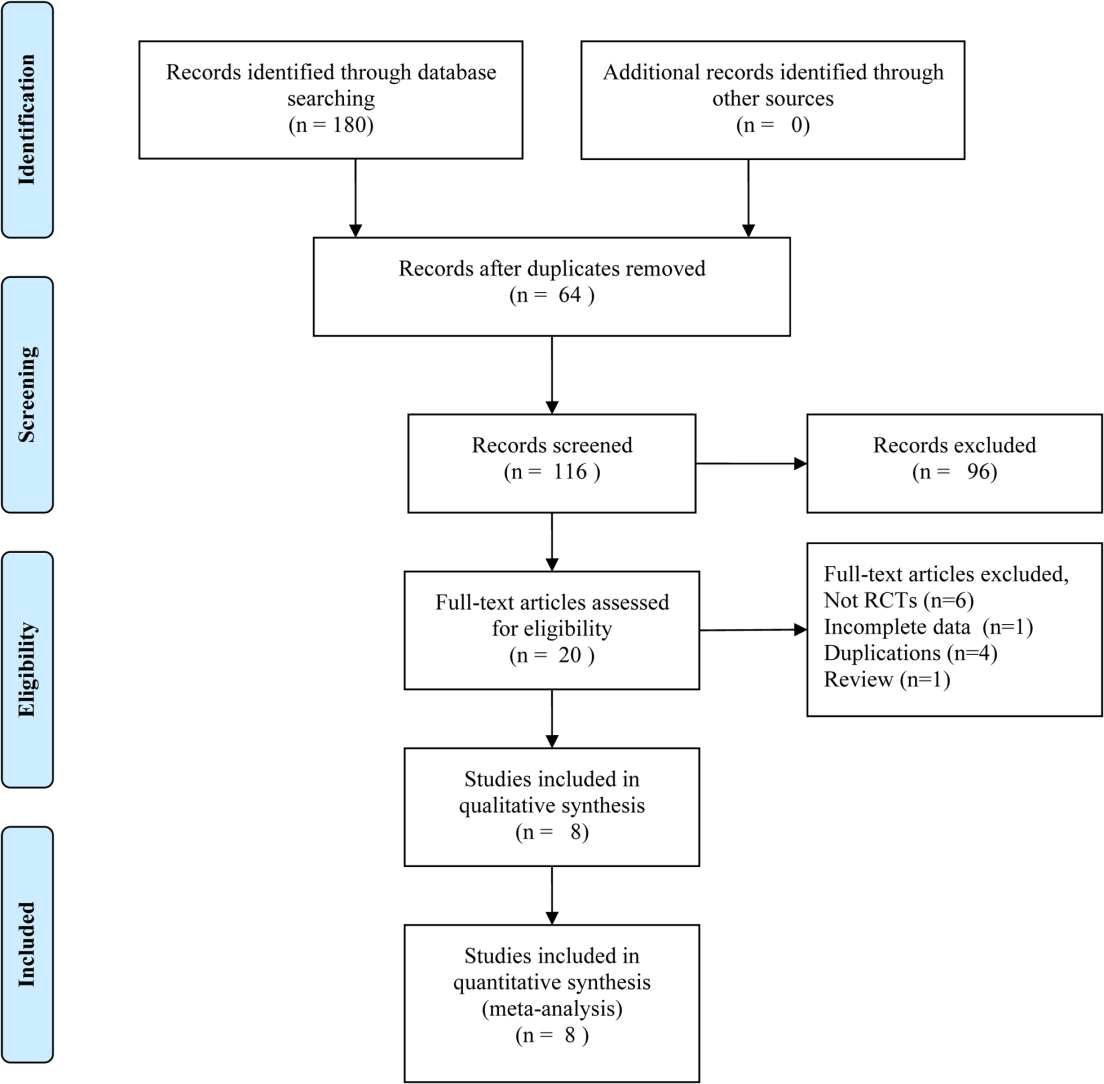
Flow diagram of literature selection.

A total of 180 records were identified according to the pre-specified search strategy. Sixty four studies were discarded by the “find duplication” function of EndNote X6 software. After screening titles and abstracts 96 studies were excluded due to not being RCTs, not Pca related, or because they were abstracts, letters and duplications. The full-text versions of the remaining 20 qualifying studies were obtained to further determine eligibility. Twelve studies were excluded due to not being RCTs (n = 6), no efficacy comparison studied (n = 1), duplications (n = 4), and one review (n = 1) (Fig 1). The remaining eight RCTs were included in the meta-analysis, involving 6,050 patients. Three RCTs for zibotentan vs. placebo [12–14], two for zibotentan plus docetaxel vs. docetaxel alone [15,16], one for atrasentan plus docetaxel vs. docetaxel alone [17], and one for atrasentan vs. placebo [18,19]. The baseline characteristics of the included studies are shown in Table 1.

**Table 1:** The characteristics of included studies.

Study	Journal	Multicenter?	Arm	Sample	Median age	Median PSA (ng/mL)	Median PFS (months)	Median OS (months)	Patients	No. death
Nelson JB 2012	Cancer	Yes	zibotentan 10mg/day	299	71.0 (46.0–90.0)	52.9 (0.7–1860.0)	6.2	24.5	Metastatic	136
			placebo	295	71.0 (46.0–95.0)	52.6 (0.1–5172.0)	6.5	22.5		147
Miller K 2013	Prostate Cancer and Prostatic Disease	Yes	zibotentan 10mg/day	703	73.0 (44.0–93.0)	NR	NR	NR	Non-metastatic	40
			placebo	712	73.0 (44.0–93.0)					39
James ND 2010	BJU International	Yes	zibotentan 10mg/day	107	70.0 (53.0–85.0)	55.0 (2.1–3725.0)		23.5	Metastatic	74
			placebo	107	72.0 (49.0–91.0)	64.0 (5.3–3776.0)		19.9		68
Fizazi K 2013	Journal of Clinical Oncology	Yes	zibotentan 10mg/day+docetaxel 75 mg/m2	524	68.0 (42.0–90.0)	83.8	NR	NR	Metastatic	277
			docetaxel 75 mg/m2	528	68.0 (40.0–86.0)	101				280
Trump DL 2011	Prostate	Yes	zibotentan 10mg/day+docetaxel 75 mg/m2	20	66.9 (52.0–84.0)	NR	NR	NR	Metastatic	NR
			docetaxel 75 mg/m2	11	70.7 (56.0–85.0)					NR
Quinn DI 2013	Lancet Oncol	Yes	atrasentan 10mg/day+docetaxel 75 mg/m2	498	69 (40–92)	79.0 (23.5–228.3)	9.2 (8.5–9.9)	17.8 (16.4–19.8)	Metastatic	3
			docetaxel 75 mg/m2	496	69 (43.0–89.0)	67.7 (24.6–202.4)	9.1 (8.4–10.2)	17.6 (16.4–20.1)		7
Carducci MA 2007	Cancer	Yes	atrasentan 10mg/day	408	73.0 (45.0–93.0)	69.8 (1.7–5784.0)	NR	20.5	Metastatic	25
			placebo	401	72.0 (45.0–92.0)	79.6 (2.2–5424.8)		20.3		21
Nelson JB 2008	Cancer	Yes	atrasentan 10mg/day	467	75.0 (47.0–92.0)	13.1 (1.2–732.9)	17.7	49.2	Non-metastatic	22
			placebo	474	74.0 (48.0–93.0)	13.1 (0.8–672.2)	21.3	46.8		21

NR, not report; PSA, prostate specific antigen; PFS, progression-free survival; OS, overall survival.

### Methodological quality assessment

The methodological quality of included studies was high. All included RCTs were conducted using a multicenter, randomized, double-blind, comparative design. Two studies [16,18] demonstrated bias. All studies addressed the complete outcome data and there was no selective reporting bias.

### Results of meta-analysis

#### Overall Survival

**Fig 2:**
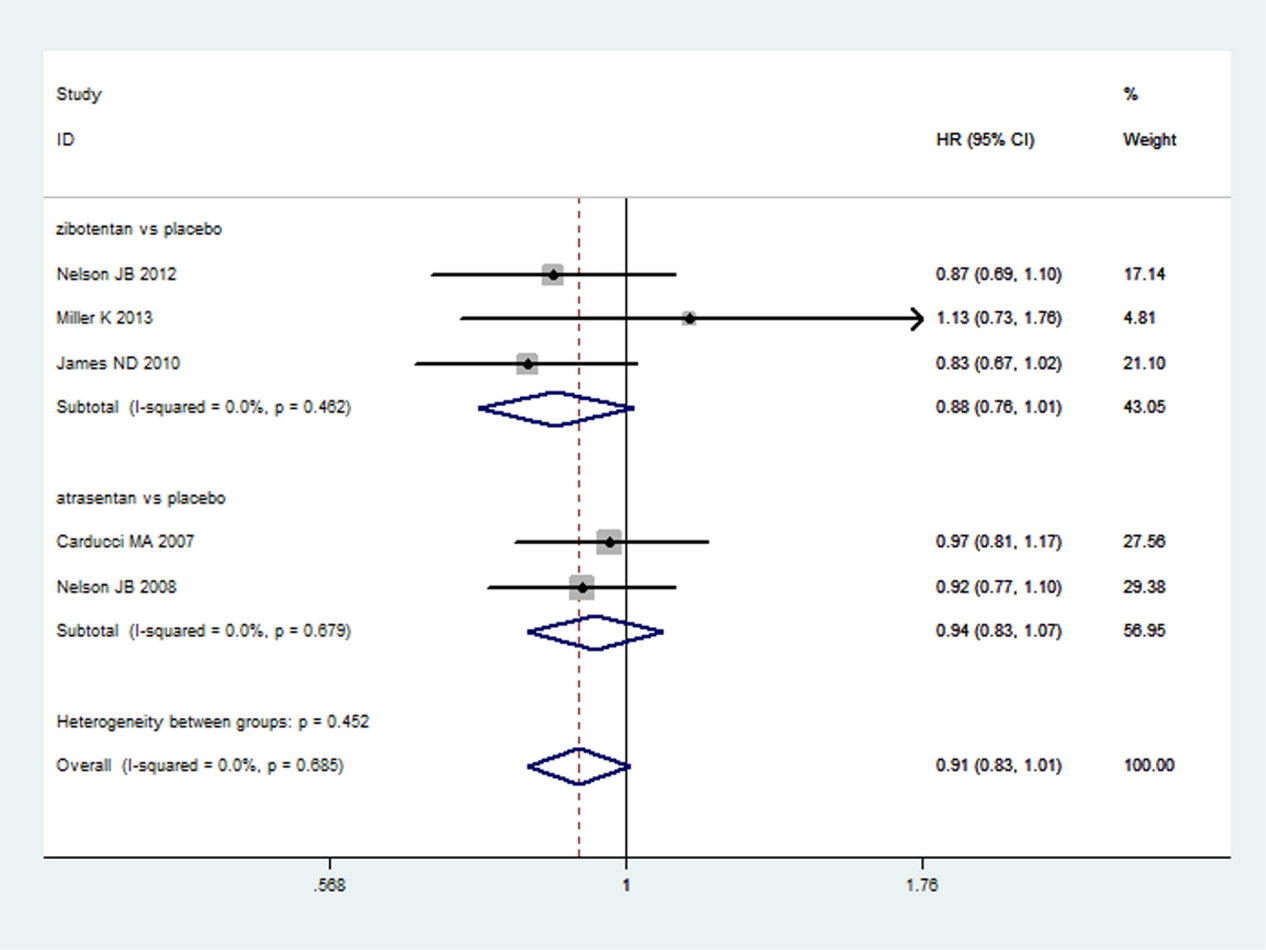
The results of direct comparison of zibotentan and atrasentan for overall survival. Squares indicate study-specific hazard ratios (size of the square reflects the study-specific statistical weight, i.e., the inverse of the variance); horizontal lines indicate 95% confidence intervals (CIs); diamonds indicate summary hazard ratios with its corresponding 95% confidence interval.

Five studies [12–14,18,19] reported OS of ET-A receptor antagonists versus placebo comparisons in a total of 3,973 patients. The heterogeneity between the five studies was P = 0.685, I^2^ = 0.0%. A fixed effect model was used to analyze the results. Compared with placebo, ET-A receptor antagonists could not significantly prolong the OS (HR = 0.91, 95%CI: 0.83–1.01; P = 0.066) (Fig 2). Two studies [15,17] reported OS of zibotentan/atrasentan plus docetaxel versus docetaxel alone, involving 2,046 patients. There was no statistical heterogeneity between the included two studies (P = 0.727, I^2^ = 0.0%). A fixed effect model was used to pool the results. ET-A receptor antagonists plus docetaxel could not significantly prolong the OS of CRPC (HR = 1.02, 95%CI: 0.92–1.14; P = 0.671) when compared with docetaxel alone (S1 File).

**Fig 3:**
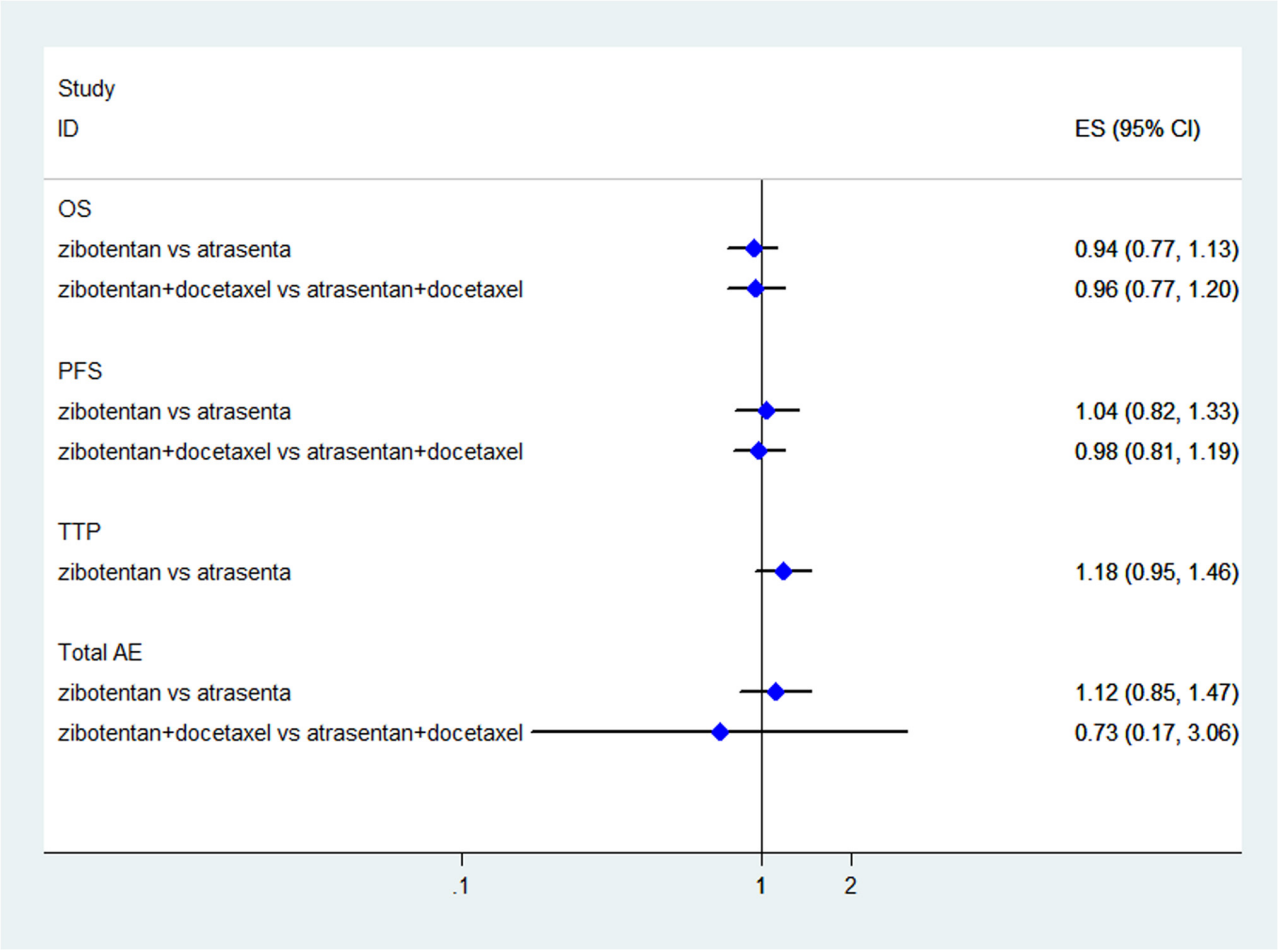
The results of indirect comparisons for OS, PFS, TTP, and total AE. Diamonds indicate summary efficacy estimates; horizontal lines indicate 95% confidence intervals (CIs).

The results of indirect comparisons showed that there were no statistically significant differences for zibotentan plus docetaxel versus atrasentan plus docetaxel compared to docotaxel alone (HR = 0.96, 95%CI: 0.77–1.20; P = 0.718) and zibotentan versus atrasentan compared with placebo (HR = 0.94, 95%CI: 0.77–1.13; P = 0.527) in the improvement of OS (Fig 3).

#### Progression-free survival

**Fig 4:**
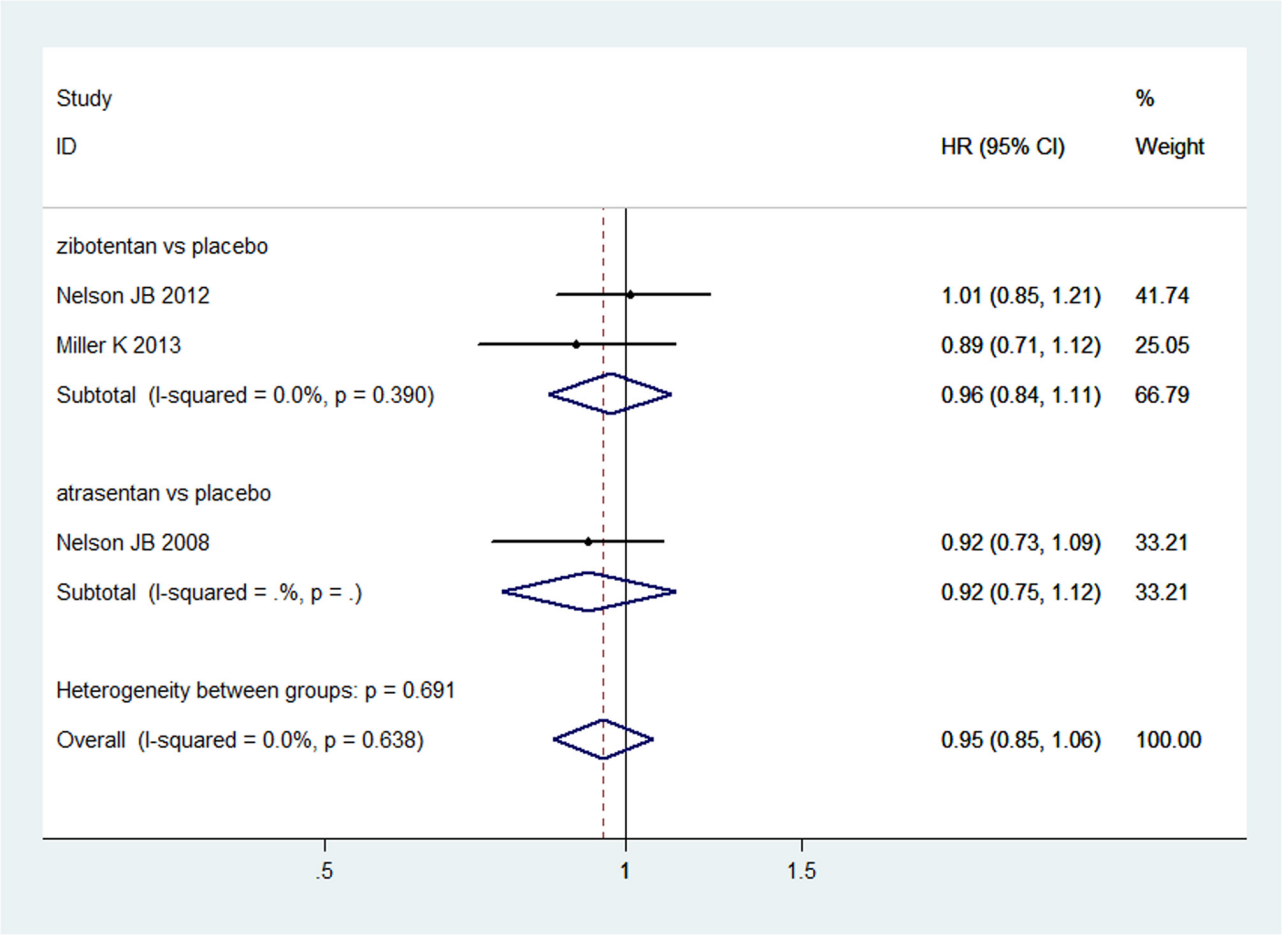
The results of direct comparison of zibotentan and atrasentan for progression-free survival. Squares indicate study-specific hazard ratios (size of the square reflects the study-specific statistical weight, i.e., the inverse of the variance); horizontal lines indicate 95% confidence intervals (CIs); diamonds indicate summary hazard ratios with its corresponding 95% confidence interval.

Three studies [12,13,19] reported PFS of ET-A receptor antagonists versus placebo. The results of heterogeneity and meta-analysis are shown in Fig 4. ET-A receptor antagonists did not significantly improve the PFS (HR = 0.95, 95%CI: 0.85–1.06; P = 0.355) compared with placebo. Two studies [15,17] reported PFS of ET-A receptor antagonists plus docetaxel versus docetaxel alone. The heterogeneity between five studies was P = 0.838, I^2^ = 0.0%. A fixed effect model was used to analyze the results. There were no statistically significant differences for the two groups (HR = 1.01, 95%CI: 0.92–1.11; P = 0.834) in the improvement of PFS (S1 File).

An indirect comparison was performed to compare the PFS of zibotentan plus docetaxel versus atrasentan plus docetaxel to docotaxel alone (HR = 0.98, 95%CI: 0.81–1.19; P = 0.837) and zibotentan versus atrasentan with placebo (HR = 1.04, 95%CI: 0.82–1.33; P = 0.751). The differences were not statistically significant (Fig 3).

#### Time to disease progression

Three studies [14,18,19] involving 1,964 patients reported TTP of ET-A receptor antagonists versus placebo. There was no statistical heterogeneity between the included three studies (P = 0.324, I^2^ = 11.3%). A fixed effect model was used to pool the results. Compared with placebo alone, ET-A receptor antagonists could not significantly prolong the TTP (HR = 0.95, 95%CI: 0.86–1.05; P = 0.285) (S1 File).

The results of indirect comparisons with placebo showed that zibotentan could not significantly prolong the TTP when compared with atrasentan (HR = 1.18, 95%CI: 0.95–1.46; P = 0.131) (Fig 3).

#### Adverse events (III-IV)

Seven studies [12,13,15–19] reported the incidences of AEs. Compared with placebo, ET-A receptor antagonists could not significantly reduce the incidence of total AE (III-IV) (OR = 1.06, 95%CI: 0.92–1.21; P = 0.418) (S1 File) or the incidence of anemia (III-IV) (OR = 1.27, 95%CI: 0.84–1.91; P = 0.258) (S1 File). On the contrary, ET-A receptor antagonists did increase the risk of headache (III-IV) (OR = 13.81, 95%CI: 2.63–72.49; P = 0.002) (S1 File) and peripheral edema (III-IV) (OR = 4.46, 95%CI: 1.03–19.24; P = 0.045) (S1 File). The results of indirect comparison showed that there were no statistically significant differences for zibotentan versus atrasentan compared with placebo in the reduction of total AE (III-IV) (OR = 1.12, 95%CI: 0.85–1.47; P = 0.417) (Fig 3).

Compared with docetaxel alone, there were no statistically significant differences for ET-A receptor antagonists plus docetaxel in the reduction of total AE (III-IV) (OR = 0.90, 95%CI: 0.68–1.18; P = 0.442) (S1 File). And zibotentan+docetaxel was not better than atrasentan+docetaxel in the reduction of total AE (III-IV) (OR = 0.73, 95%CI: 0.17–3.06; P = 0.670) (Fig 3). Moreover, ET-A receptor antagonists plus docetaxel could not reduce the incidence of leukopenia (III-IV) (OR = 0.97, 95%CI: 0.63–1.49; P = 0.895) (S1 File) and neutropenia (III-IV) (OR = 0.85, 95%CI: 0.62–1.17; P = 0.319) (S1 File).

### Publication Bias

**Fig 5:**
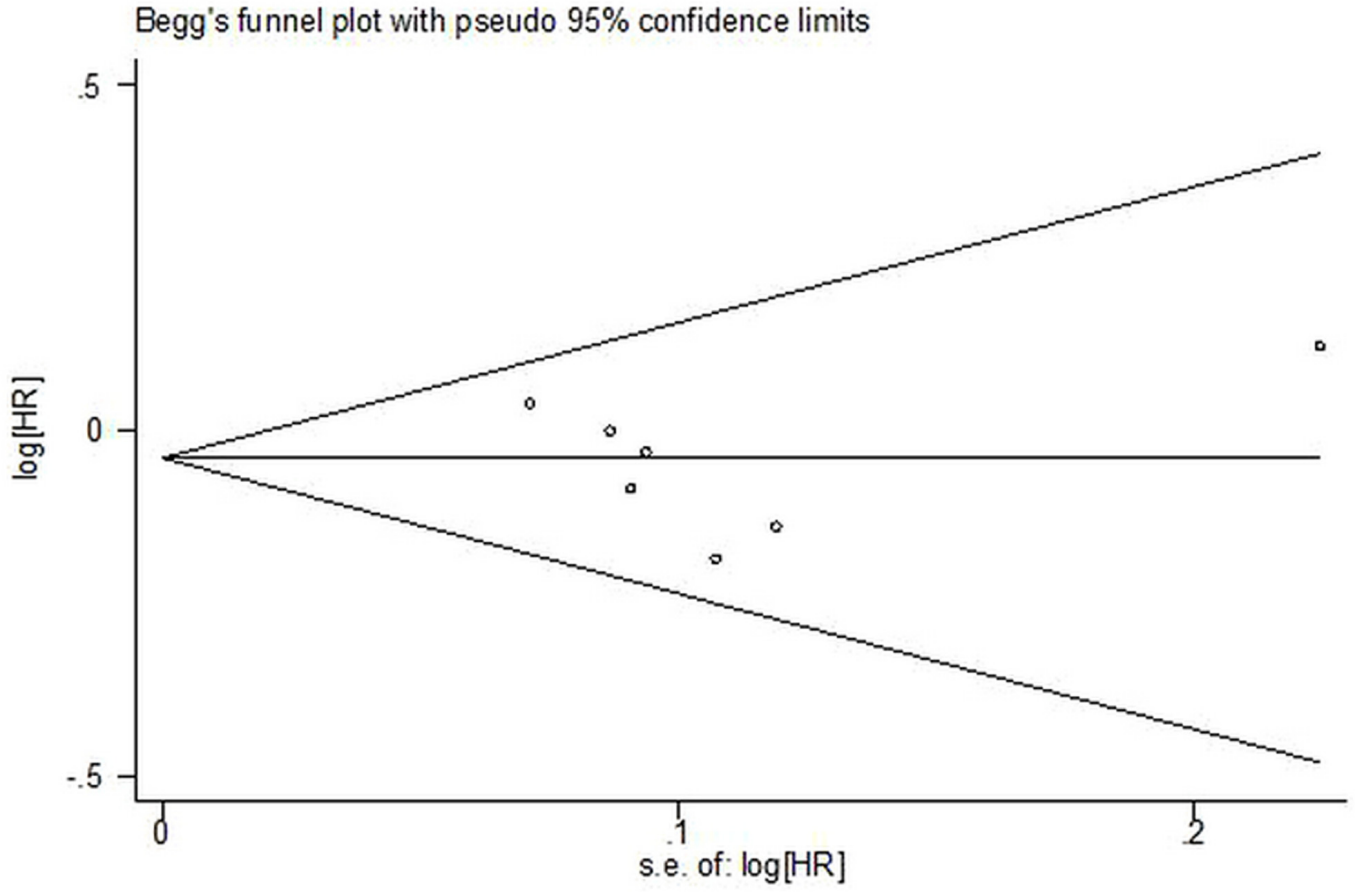
Assessment of publication bias.

In our study there was no publication bias found. First, a comprehensive literature search was conducted and there were not any limitations such as language. Secondly, the funnel plot was performed to identify publication bias and none was found. (Begg's test, p = 0.368; Egger's test, p = 0.679, See Fig 5).

### Sensitivity analysis

**Fig 6:**
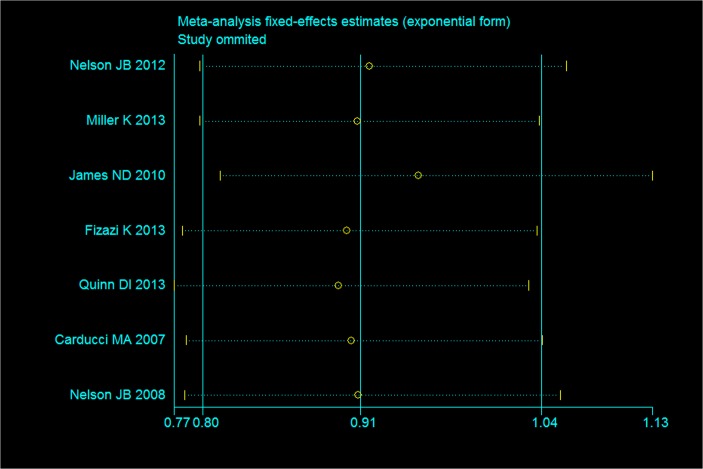
Assessment of sensitivity analysis for OS.

Fig 6 shows the results of sensitivity analysis regarding OS. The result indicates that excluded studies did not influence the overall effective size.

## Discussion

### Summary of findings

This is a comprehensive meta-analysis to directly compare the efficacy of ET-A receptor antagonists with placebo or docetaxel in CRPC patients. More importantly, an indirect comparison was performed primarily to compare the efficacy between zibotentan and atrasentan as opposed to placebo due to the lack of direct comparison evidence. The results of direct comparison showed that, compared with placebo, treatment with ET-A receptor antagonists could not improve overall survival, progression-free survival, or time to disease progression. While there was no reduction in the incidence of total AE (III-IV), more headache(III-IV) and peripheral edema (III-IV) was found in the ET-A receptor antagonists group. Similarly, ET-A receptor antagonists plus docetaxel was inferior in the improvement of overall survival, progression-free survival, time to disease progression and AEs when compared with docetaxel alone. The results of indirect comparisons with placebo indicate that the effectiveness of zibotentan was equal to atrasentan. The result was similar between zibotentan plus docetaxel and atrasentan plus docetaxel compared with docetaxel alone. The indirect comparisons of ET-A receptor antagonists versus ET-A receptor antagonists plus docetaxel were not conducted due to the lack of a common comparator.

### Clinical implications

The ET-A receptor antagonists have been implicated in the progression of CRPC. The initial results of a phase 2 trial involving 312 patients showed that a signal for prolonged overall survival was observed in the ET-A receptor antagonist treatment group versus placebo, and it was well tolerated [20]. However, a phase 3 trial involving 594 patients conducted by Nelson [12] showed that the treatment with zibotentan did not lead to a statistically significant improvement in OS. Similar disagreements could be found in other original studies [13,15]. A meta-analysis was needed to confirm the efficacy of ET-A receptor antagonists in the treatment of CRPC. And an indirect comparison was also needed to compare the differences between ET-A receptor antagonists because there were no head-to-head RCTs or meta-analyses. Our results of direct and indirect comparisons showed that ET-A receptor antagonists with or without docetaxel could not lead to significant improvements in the outcomes of effectiveness and safety when compared with placebo or docetaxel. Therefore, single-agent docetaxel should remain as one of the standard treatments. Obviously, the negative results were found in our meta-analysis. The reasons for negative results might be inadequately statistical power and the sample size differences of included studies (the range of sample size: 31–1,415). More high quality RCTs are still needed to confirm our results, and combination regimens based on ET-A receptor antagonists should be explored for CRPC in future research.

### Strengths and limitations

This is the first indirect comparison of ET-A receptor antagonists for CRPC. The results indicate that there are no differences between ET-A receptor antagonists for CRPC, which resolves the difficulty of the lack of head-to-head studies. This is the first systematic meta-analysis to demonstrate the efficacy of ET-A receptor antagonists for CRPC. Moreover, the methodological quality of the included RCTs was good.

Some limitations were found in our study however. First, only eight RCTs were identified through systematic literature search including common literature databases and other sources. A publication bias was not observed according to Begg’s funnel plot. Second, subgroup analysis of metastatic and non-metastatic patients was not conducted due to limited data. In addition, the indirect comparisons of zibotentan/atrasentan versus zibotentan/atrasentan plus docetaxel were not conducted due to the lack of a common comparator. Future studies about zibotentan/atrasentan versus docetaxel for CRPC are needed.

## Conclusions

Our evidence indicates that there are no significant benefits for ET-A receptor antagonists with or without docetaxel with regard to PFS, OS, TTP, and overall AEs. There is no significant difference in efficacy between zibotentan and atrasentan. Single-agent docetaxel should remain as one of the standard treatments. More phase III clinical trials which assess the efficacy of ET-A receptor antagonists for CRPC are needed. Direct comparison RCTs of ET-A receptor antagonists versus ET-A receptor antagonists plus docetaxel are also needed to confirm the efficacy and safety of ET-A receptor antagonists.

## Supporting Information

S1 PRISMA Checklist PRISMA Checklist (DOC)

S1 File The results of direct comparisons. (PDF)
