# Supplementary material for: A Meta-Analysis and Indirect Comparison of Endothelin A Receptor Antagonist for Castration-Resistant Prostate Cancer
Source: PLoS One. 2015 Jul 20;10(7):e0133803. doi: 10.1371/journal.pone.0133803 (PMC4508042; doi:10.1371/journal.pone.0133803)
Supplement: S1 File — (PDF) [file pone.0133803.s002.pdf]

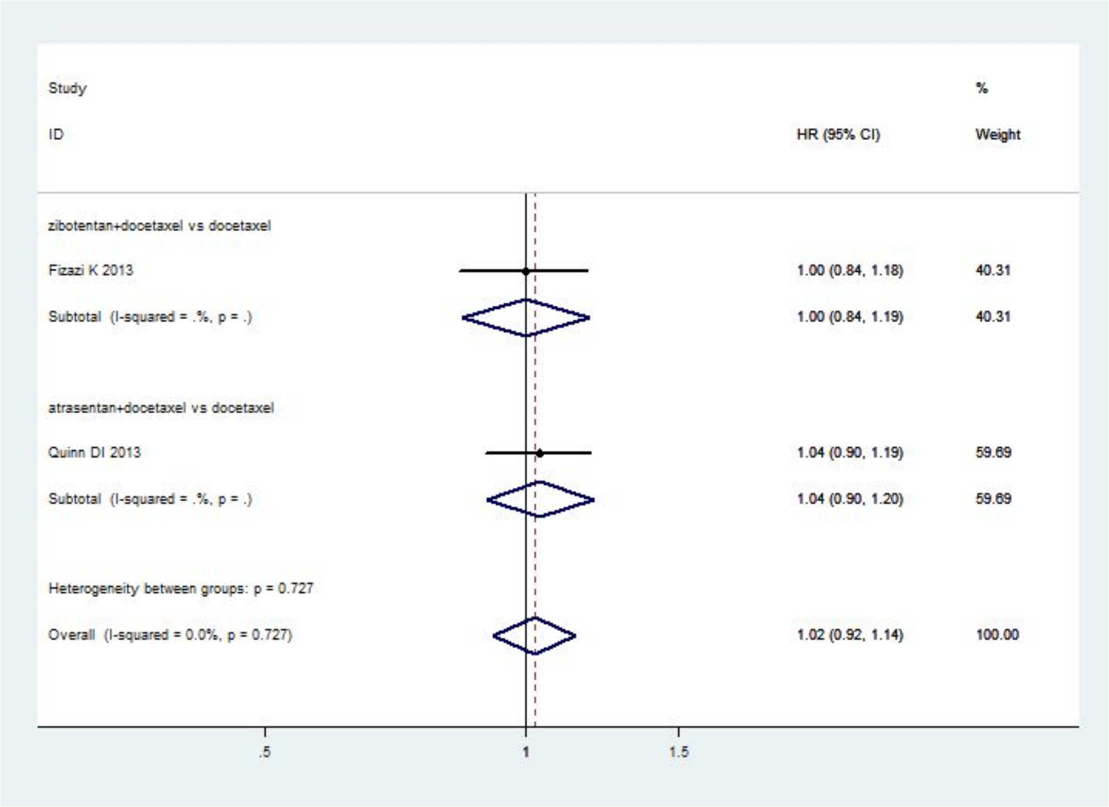

S 1-1 The results of OS of ET-A receptor antagonists plus docetaxel versus docetaxel alone

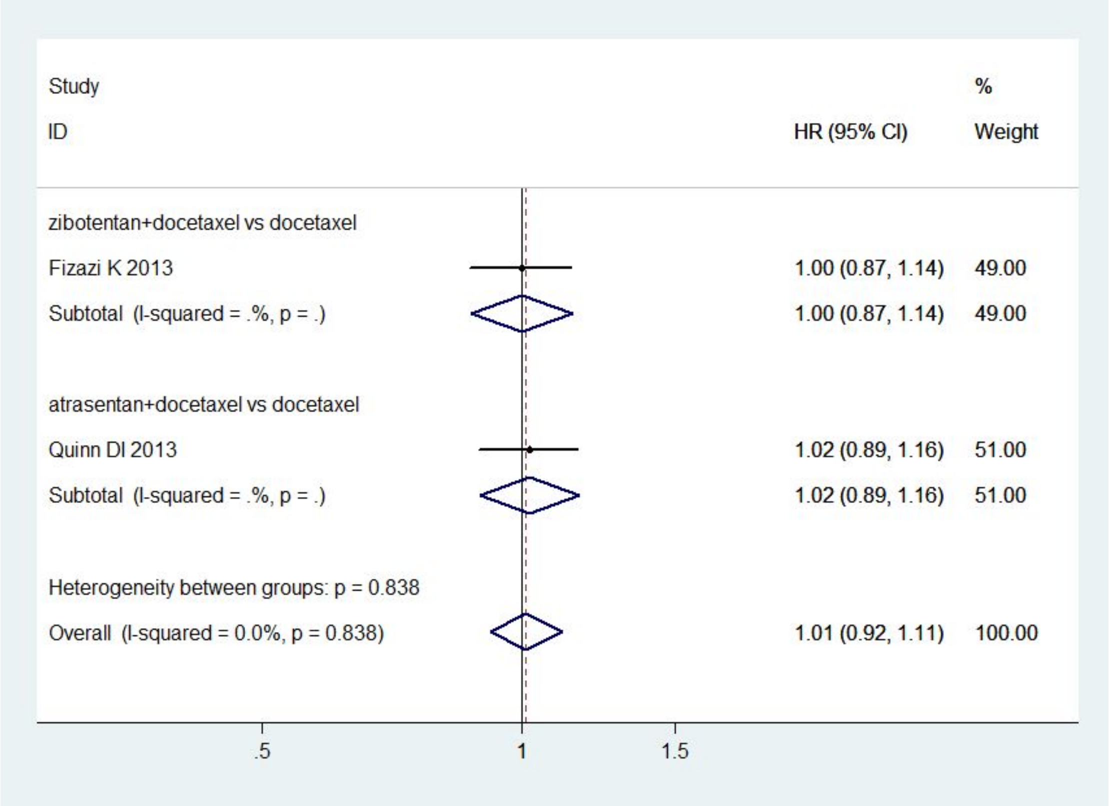

S 1-2 The results of PFS of ET-A receptor antagonists plus docetaxel versus docetaxel alone

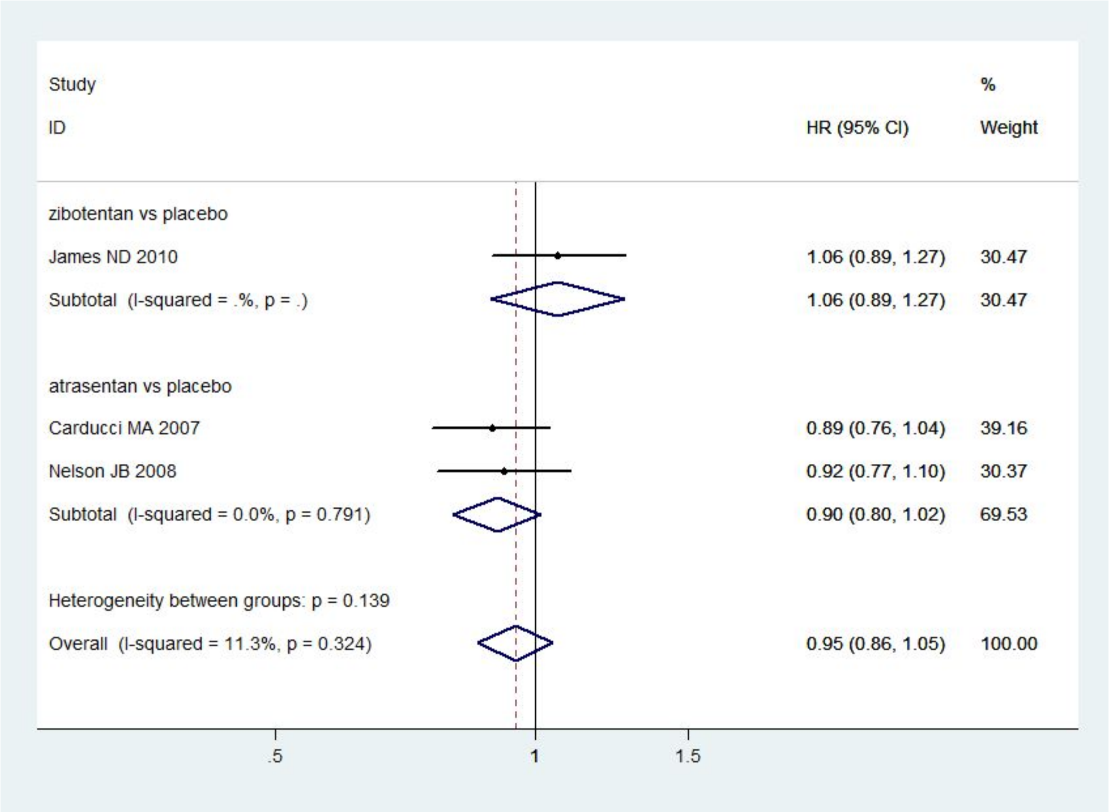

S 1-3 The results of direct comparison of time to disease progression

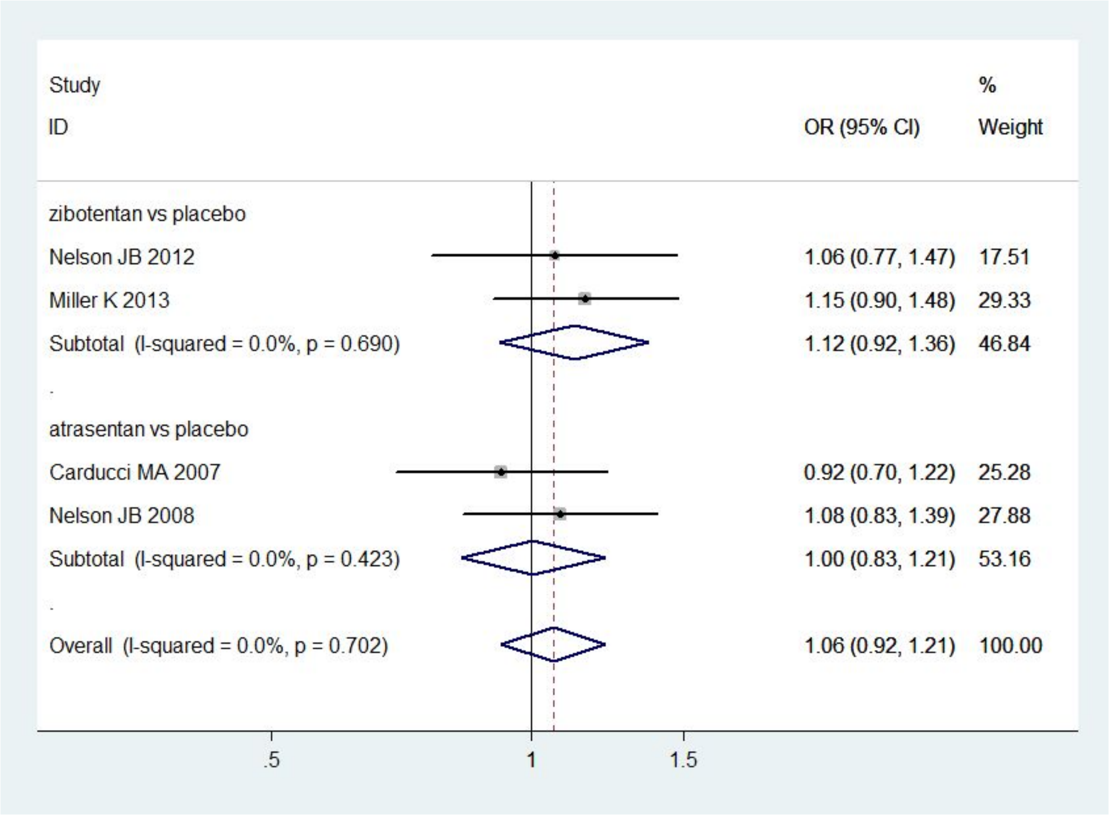

S 1-4 The results of direct comparison of total adverse events (III-IV) for ET-A receptor antagonists versus placebo

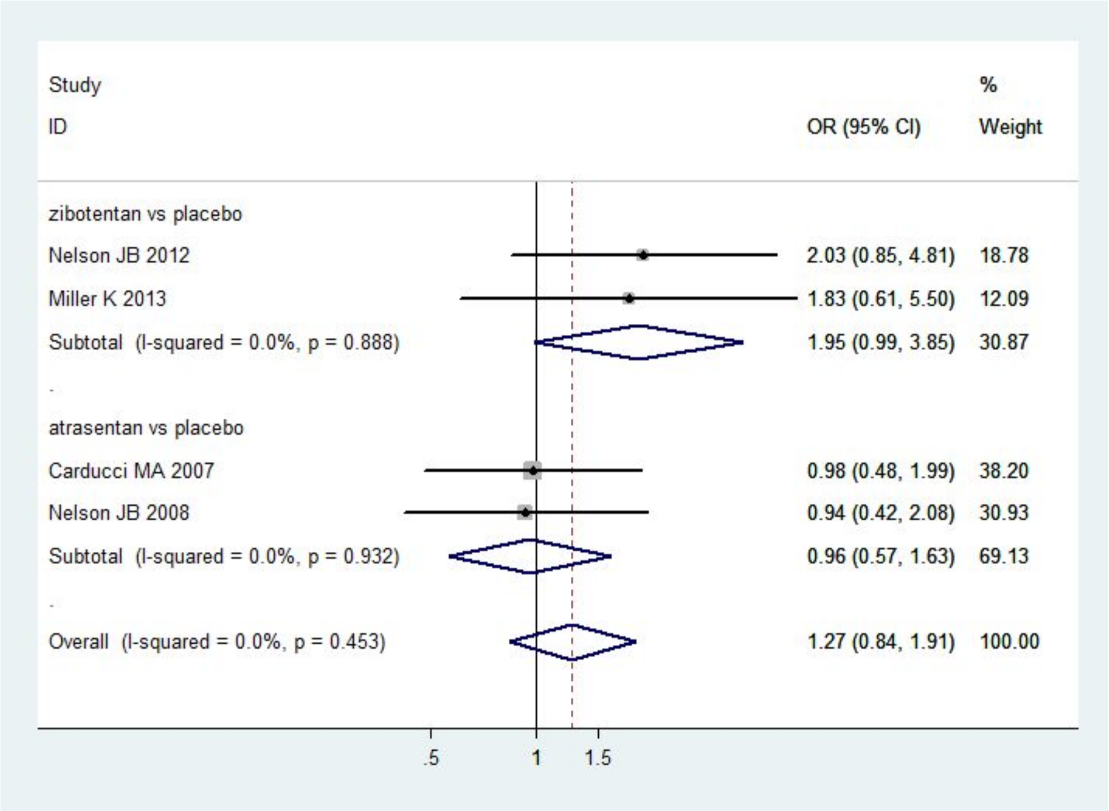

S 1-5 The results of anemia (III-IV) of ET-A receptor antagonists versus placebo

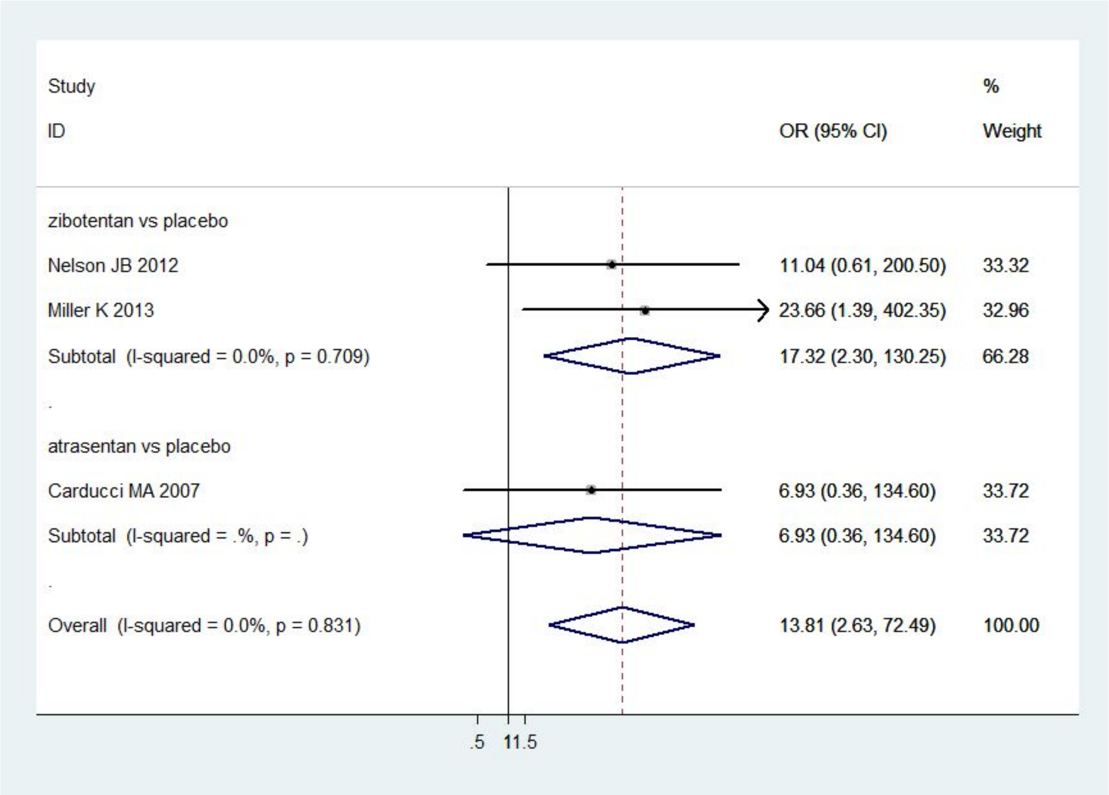

S 1-6 The results of headache (III-IV) of ET-A receptor antagonists versus placebo

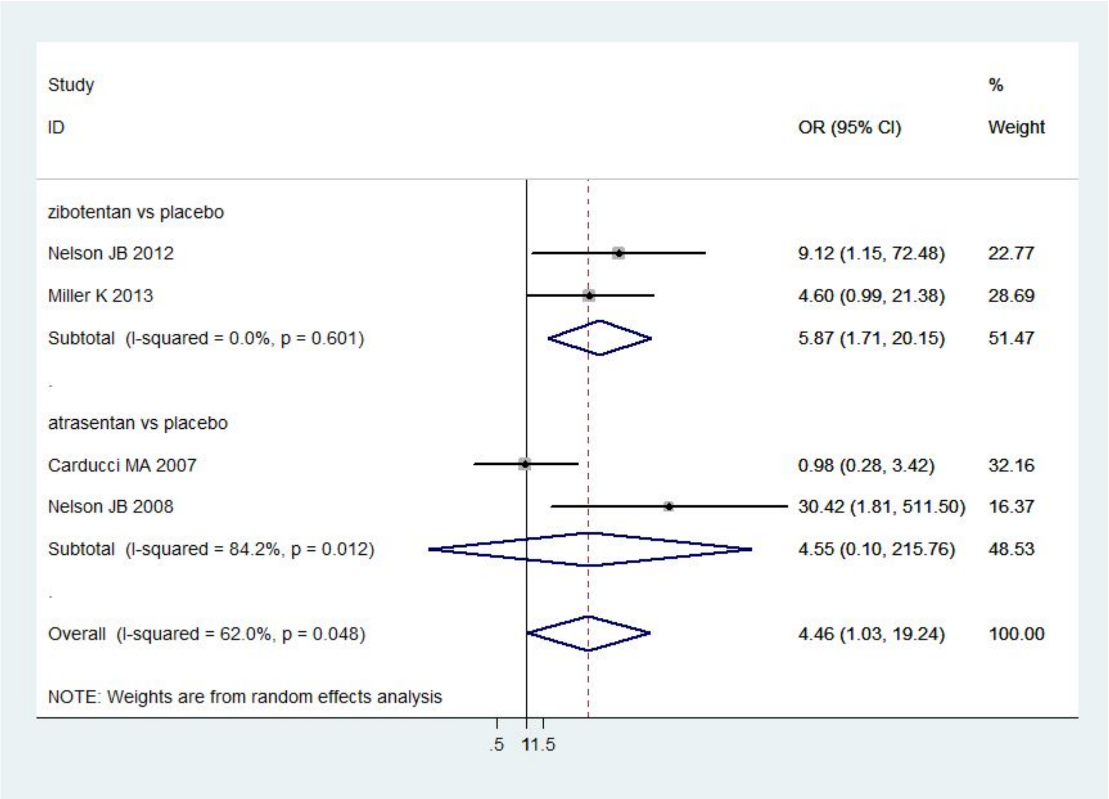

S 1-7 The results of peripheral oedema (III-IV) of ET-A receptor antagonists versus placebo

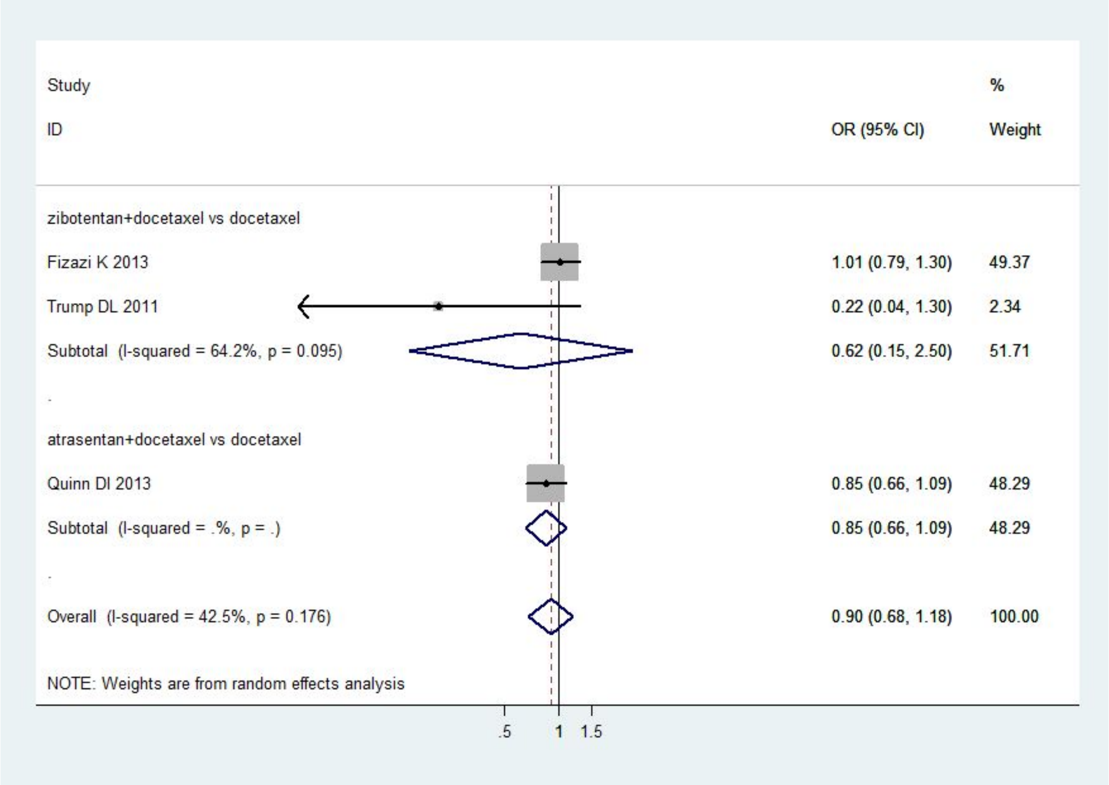

S 1-8 The results of direct comparison of total adverse events (III-IV) for ET-A receptor antagonists plus docetaxel

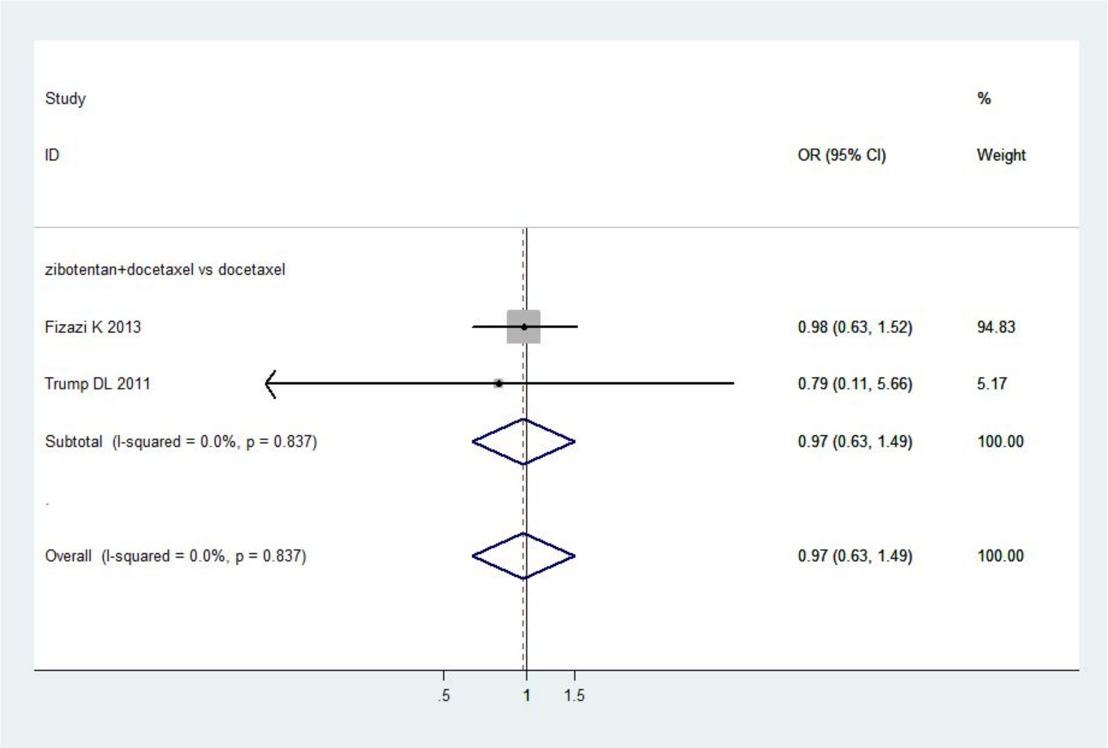

S 1-9 The results of leukopenia (III-IV) of ET-A receptor antagonists plus docetaxel versus docetaxel alone

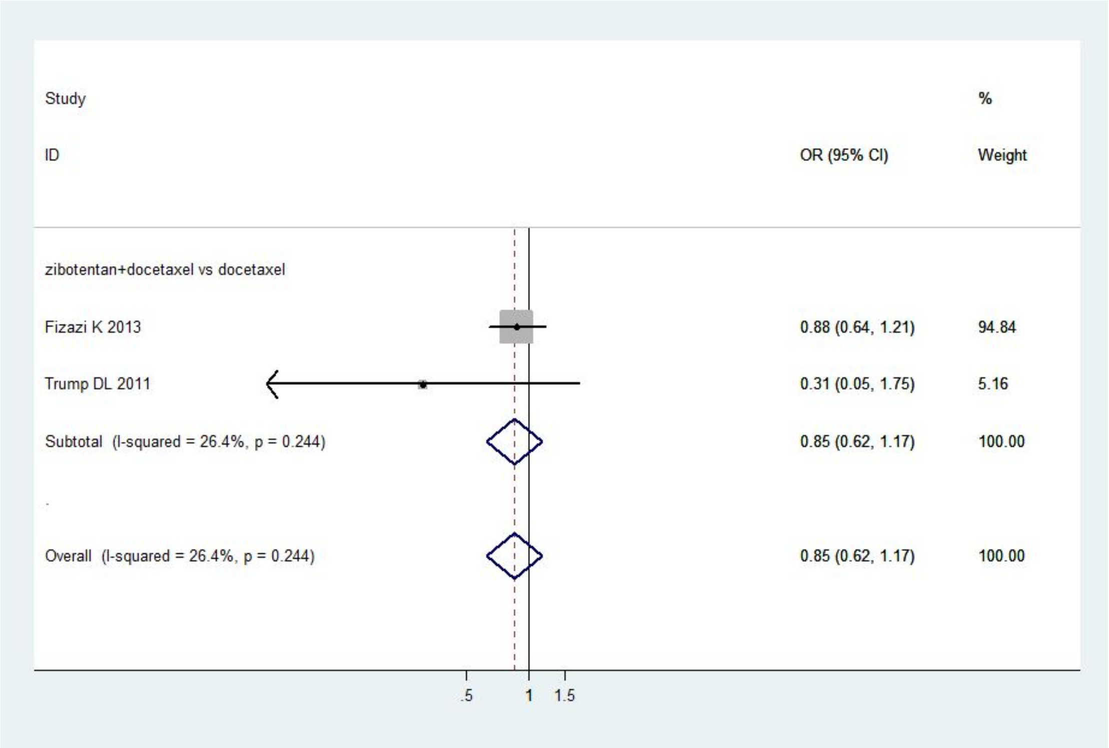

S 1-10 The results of neutropenia (III-IV) of ET-A receptor antagonists plus docetaxel versus docetaxel alone
